# The rising incidence of stroke in the young: Epidemiology, causes and global impact

**DOI:** 10.1177/17474930251362583

**Published:** 2025-07-18

**Authors:** Ahmad Nehme, Linxin Li

**Affiliations:** Wolfson Centre For Prevention of Stroke and Dementia, Nuffield Department of Clinical Neurosciences, John Radcliffe Hospital, University of Oxford, Oxford, UK

**Keywords:** Epidemiology, risk factors, prevention, young stroke, stroke incidence, burden

## Abstract

Although reductions in stroke incidence have been reported over recent decades particularly in high-income countries, there has been a worrying trend since the start of the 21st century: stroke incidence in younger individuals (<55 years) has not showed a similar decrease as at older ages. In high-income countries, several population-based studies have found an increase in the incidence of stroke at younger ages since 2000, reaching up to 90% in Oxfordshire, UK (2010–2018 vs 1981–1986) and 97% in Cincinnati, USA (2010 vs 1993–1994). A similar picture has also been documented in low- and middle-income countries, both in population-based studies (Joinville, Brazil, 35% increase in 2012–2013 vs 2005–2006) and in the Global Burden of Disease study. The exact reasons for this very different picture seen in younger individuals are unknown. One possibility is that traditional modifiable risk factors are increasingly prevalent and often undertreated at younger ages. However, studies have also found increases in the incidence of young-onset cryptogenic stroke and in people with no traditional modifiable risk factors, suggesting a role for emerging risk factors. Potential culprits might include air pollution, long working hours, psychosocial stress, prior autoimmune diseases, and illicit drug use, although further research is required to determine whether these emerging risk factors are causally related to stroke at younger ages. Without further intervention, the global burden of stroke at younger ages is projected to increase further in the coming years. Therefore, there is an urgent need to better understand the drivers of these time trends in incidence, to potentially alleviate the individual and societal impacts of stroke in the young. In this narrative review, we examine the recent global changes in stroke epidemiology at younger ages, their potential drivers, and their projected consequences.

## Introduction

Stroke is traditionally considered a disease of older ages. In high-income countries (HICs), the median age at the time of a first-ever stroke is over 70 years,^1^ and only 5–15% of cases occur at younger ages (commonly defined as <55 years).^2–5^ In low- and middle-income countries (LMICs), patients with stroke are on average 5 years younger than in HICs,^6^ with a higher proportion of cases occurring at younger ages, ranging from 14% to 24%.^7–10^

Recently, multiple studies have reported an increase in stroke incidence at younger ages, which contrasts with the decrease in stroke incidence at older ages and parallels trends in early-onset cancer and mortality.^11–13^ The exact reasons for this increase are unknown, with potential contributions of traditional and emerging vascular risk factors. This increase is projected to have important public health consequences due to the economic costs of stroke at younger ages and its impacts on disability, quality of life, and premature mortality. A review of the changing landscape of stroke epidemiology at younger ages is necessary to contextualize recent trends and generate mechanistic hypotheses. In this review, we aimed to provide an up-to-date summary of recent trends in stroke incidence at younger ages as well as to discuss their potential drivers and consequences.

## Incidence: a changing landscape

**Figure 1.**
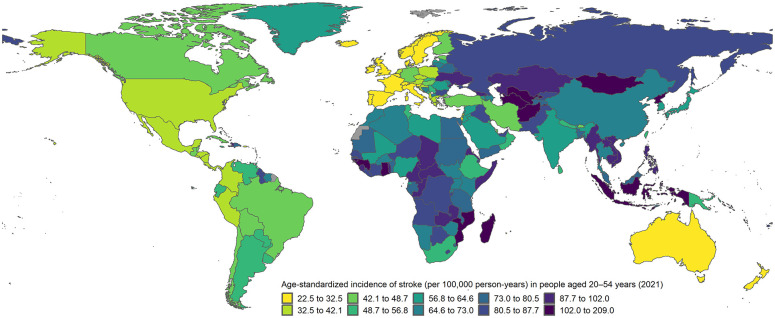
Age-standardized incidence of stroke at ages 20–54 years according to 2021 estimates from the Global Burden of Disease study. Incidence rates age-standardized by 5-year age groups to the world standard population.

The landscape of stroke incidence at younger ages has changed in the last two decades, with different trends compared to stroke at older ages and marked disparities between HICs and LMICs (Figure 1).

### HICs

Due to improvements in primary prevention, stroke incidence decreased overall at an annual rate of 1.0–1.5% in HICs between 1985 and 2017.^14^ However, this mostly resulted from a decrease at older ages. A recent systematic review found that of 36 population- or administrative-based studies reporting at least one period after 2000, more than half (n = 24) showed an increase in stroke incidence at younger ages.^13^ The rest reported stable or declining rates. Part of the heterogeneity was explained by differences in study methodology. Incidence rates increased in the five population-based studies that used constant methods to maximize the ascertainment of minor events, which make up a larger proportion of strokes at younger than at older ages.^13,15^ In these studies, the absolute increase in incidence ranged from 21% to 97%,^2,5,13,16,17^ with the most prominent rise in the United States. Few studies reported time trends stratified by etiology. In the Oxford Vascular study (OxVasc), the greatest absolute increases were in cryptogenic events and small-vessel occlusion, with stable rates of rare causes like arterial dissection.^3^ According to the 2021 Global Burden of Disease (GBD) study, the median age-standardized incidence rate (ASIR, world standard population) of stroke at ages 20–54 years in HICs was 42.0 per 100,000 person-years (interquartile range (IQR): 31.4–55.1), with the lowest rates in Luxembourg (22.5) and Italy (22.9) and the highest rates in the United Arab Emirates (81.8) and Nauru (180.7).^18^

Although time trends in absolute incidence rates were heterogeneous, the same systematic review showed a consistent age-specific divergence. Nearly all studies found that stroke incidence evolved less favorably at younger than at older ages. Even in studies where young stroke incidence decreased over time, this was to a lesser extent than at older ages (Table 1). Similar findings were identified irrespective of the definition of young stroke (<45 or <55 years), stroke subtype, sex, race, or ethnicity.^13^

**Table 1.** Time trends in stroke incidence at ages <55 years and time trends in age-specific stroke incidence at younger versus older ages in recent population-based studies.

Name	Time periods	IRR (95% CI)	RTTR (95% CI)
**North America**			
Texas, USA	2017 vs 2000	1.14 (0.82–1.59)	1.67 (1.18–2.36)
Cincinnati, Ohio, USA	2010 vs 1993–1994	1.97 (1.67–2.32)	2.62 (2.19–3.13)
**Europe**			
Oxfordshire, UK	2010–2018 vs 1981–1986	1.90 (1.37–2.65)^a^	2.63 (1.86–3.72)
South London, UK	2012–2015 vs 2000–2003	0.85 (0.63–1.14)^a^	1.60 (1.17–2.21)
Orebro, Sweden	2017 vs 1999	0.76 (0.41–1.41)	1.68 (0.89–3.20)
Lund, Sweden	2015–2016 vs 2001–2002	0.88 (0.54–1.46)	1.26 (0.75–2.11)
Dijon, France	2003–2011 vs 1985–1993	1.74 (1.39–2.19)	1.52 (1.19–1.93)
Valle d’Aosta, Italy	2004–2008 vs 1989	1.12 (0.70–1.80)	1.46 (0.89–2.40)
Porto, Portugal	2009–2011 vs 1998–2000	0.80 (0.57–1.13)	1.03 (0.71–1.50)
Arcadia, Greece	2015–2016 vs 1993–1995	1.08 (0.63–1.85)	1.07 (0.61–1.86)
Tartu, Estonia	2001–2003 vs 1991–1993	0.69 (0.47–0.99)^a^	0.74 (0.51–1.07)
**The rest of the world**			
Auckland, New Zealand	2011–2012 vs 2002–2003	1.50 (1.19–1.89)	2.10 (1.64–2.68)
Martinique, French West Indies	2011–2012 vs 1998–1999	1.21 (0.89–1.64)	1.88 (1.35–2.61)
Takashima, Japan	1999–2001 vs 1990–1992	1.19 (0.71–1.98)	1.26 (0.74–2.16)
Joinville, Brazil	2012–2013 vs 2005–2006	1.35 (1.12–1.64)	1.74 (1.52–1.96)
Matão, Brazil	2015–2016 vs 2003–2004	0.65 (0.27–1.57)	1.42 (0.53–2.31)

IRR: incidence rate ratio; RTTR: relative temporal trend ratio; IRRs were calculated at ages < 55 years, with IRR > 1 indicating an increase in the later time periods. RTTRs were calculated for younger versus older age groups with RTTR > 1 indicating a less favorable trend at younger ages.

a Age- and sex-standardized IRRs. Studies referenced for this table are listed in the Supplemental Web Appendix.

In studies where young stroke incidence increased, this mainly occurred during the first decade of the 21st century.^13^ Recent population-based studies suggested that incidence rates may have stabilized after 2010 but not returned to their previous level.^3,16,19^ The potential impact of the COVID-19 pandemic on young stroke incidence is unknown, with more difficult ascertainment of minor events, potential setbacks in primary prevention, and the occurrence of young stroke cases due to SARS-CoV-2 infection.^20^

### LMICs

The burden and features of young stroke differ in LMICs compared to HICs. In the INTERSTROKE study, the proportion of patients aged ⩽45 years was higher in countries from Africa (21%) or Southeast Asia (18%) compared to Western Europe, North America or Australia (7%).^21^ The distribution of young stroke subtypes also varied by region, with a larger proportion of intracerebral hemorrhage (ICH) in South America (46% of all strokes), Southeast Asia (39%), or Africa (32%) compared to Western Europe, North America, or Australia (5%).^22^

Young stroke incidence rates are generally higher in LMICs than in HICs. A population-based study from Iquique (Chile) found that the ASIR of stroke at ages < 54 years was 20.7 per 100,000 person-years in 2000–2002.^7^ In China, a nationwide survey in 2013 estimated an ASIR of stroke at ages 20–49 years of 42.4 per 100,000 person-years.^23^ In a population-based study from Tanzania (2003–2006), the ASIR of stroke at ages < 54 years was substantially higher at 79.8 per 100,000 person-years, highlighting important inequalities between countries.^8^ The GBD study suggested these disparities partly reflect differences in social and economic development. Stratifying 2021 GBD estimates by socio-demographic index (SDI), the ASIR of stroke at ages 20–54 years were 44.8 (high SDI), 66.6 (middle SDI), and 73.4 (low SDI) per 100,000 person-years.^18^

**Figure 2.**
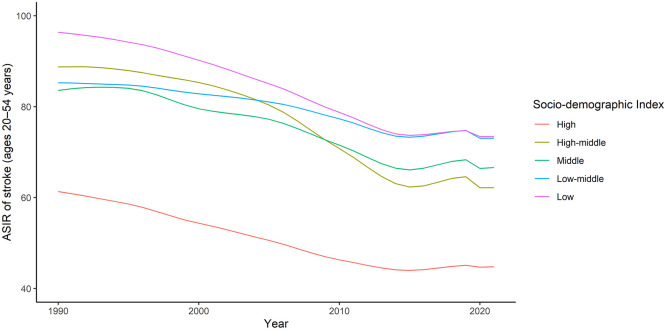
Time trends in age-standardized incidence of stroke at ages 20–54 years according to the Global Burden of Disease study (1990–2021), stratified by socio-demographic index. Incidence rates age-standardized by 5-year age groups to the world standard population and reported per 100,000 person-years. ASIR: age-standardized incidence rate; SDI: socio-demographic index.

Unfavorable time trends in young stroke incidence have also been identified in LMICs. Globally, the GBD study found that the ASIR of stroke at ages 20–54 years decreased between 1990 and 2015 but then mildly increased between 2015 and 2019 (Figure 2).^18^ This reversal was more pronounced for ischemic stroke and in countries with a high-middle or middle SDI. There was also a hint that young stroke incidence may have decreased between 2019 and 2021. Further data are needed to determine whether this was a true reduction or the result of under-ascertainment during the COVID-19 pandemic.

At a country level, few population-based studies from LMICs have reported time trends in young stroke incidence.^10,24–28^ In Joinville (Brazil), stroke incidence increased by 88% at ages < 45 years from 2005–2006 to 2010–2011, with subsequently stable rates in 2014–2015.^26^ This increase concerned ischemic stroke and ICH, was more pronounced for small-vessel occlusion, and contrasted with a decrease in stroke incidence at older ages.^26,29^ In Tianjin (China), stroke incidence at ages 35–54 years increased by twofold to threefold between 1992 and 2015.^27^ Other population-based studies have reported conflicting results. In Matão (Brazil), young stroke incidence possibly decreased in 2015–2016 compared to 2003–2004, but only 23 events were recorded across both time periods.^10^ Interestingly, a divergence by age was still found, confirming a less favorable trend at younger compared to older ages. In Tartu (Estonia), young stroke incidence decreased in 2013–2017 and 2001–2003 compared to 1991–1993, with no apparent age-specific divergence.^28^ However, young stroke incidence was much higher in Estonia than in other European HICs in 1991–1993. This decline in incidence coincided with a period of important economic growth, with Estonia classified as a HIC by the World Bank since 2006. These changes could have improved primary prevention, causing stroke incidence rates at younger ages in Estonia to decrease closer to the level of other European HICs.

## Mechanisms: measurement artifact or true increase in incidence?

Given the alarming increase in stroke incidence at younger ages, there is a crucial need to better understand its main drivers, with several possible hypotheses.

### Chance or artifact?

The consistently unfavorable time trends in young stroke incidence across studies make a chance finding unlikely, with an age-specific divergence found in countries that have markedly different healthcare systems, methods of measuring incidence, and distribution of stroke subtypes.

In HICs, the increased incidence in administrative or registry-based studies could have resulted from improved coding of stroke over time or more frequent hospital management of young patients.^30^ However, similar time trends in incidence were found in population-based studies that used constant methods of ascertainment and included patients diagnosed in all settings. Even though ischemic stroke has transitioned from a time- to a tissue-based definition, population-based studies continued using the time-based definition in recent time periods. Moreover, several studies found an increase in the incidence of both transient ischemic attack (TIA) and stroke at younger ages, suggesting that the observed time trends did not result from a diagnostic drift from TIA to stroke.^3,13^ Identification of strokes may have improved due to better recognition of stroke symptoms and more widespread use of magnetic resonance imaging (MRI). However, these changes were not specific to young people. Moreover, the Greater Cincinnati Northern Kentucky study showed that the use of MRI may not substantially change stroke incidence estimates, as incorporating MRI findings “rules out” almost the same number of strokes as it “rules in.”^31^ Furthermore, the increase in incidence at younger ages was not limited to minor ischemic events, with OxVasc finding increased rates of major or disabling stroke and ICH in 2010–2018 compared to 2002–2010.^3^

In LMICs, there have been few ideal population-based studies with consistent and near-complete ascertainment of stroke over time. Consequently, trends in young stroke incidence may be more difficult to interpret, as there have been important variations across settings in patient behavior, diagnostic practices, and access to neuroimaging.

### Traditional modifiable risk factors?

**Figure 3.**
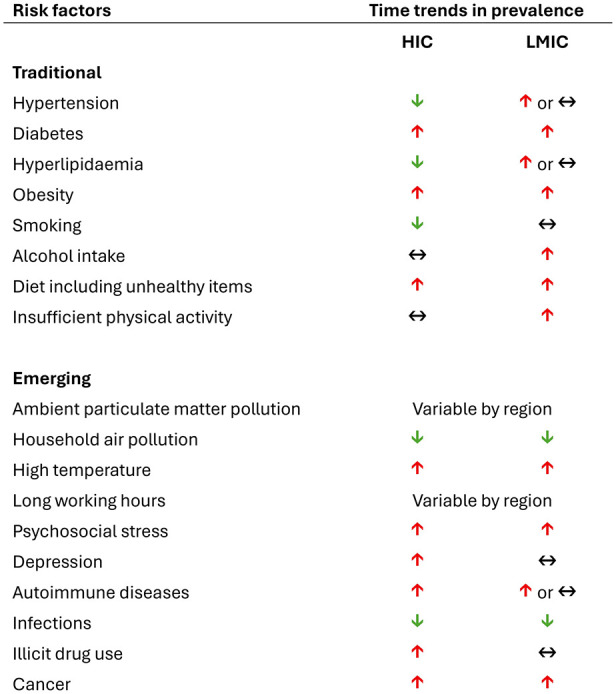
Time trends of traditional and emerging risk factors for stroke at younger ages in high-income and low- and middle-income countries. HIC: high-income countries; LMIC: low- and middle-income countries. Studies referenced for this table are listed in the Supplemental Web Appendix.

In contrast to the historical view that traditional modifiable stroke risk factors (Figure 3) are mainly relevant at older ages, their role is increasingly recognized at younger ages, as they are frequent and strongly associated with stroke in the young.^32^ In a German nationwide study, 47% of young stroke patients were current smokers, 45% had known hypertension, 31% had hyperlipidemia, 22% were obese, and 10% had diabetes.^32^ Similarly high prevalence rates were found in recent studies from the United Kingdom and the United States.^3,33^ The relative association between certain traditional modifiable risk factors and stroke tends to be stronger at younger compared to older ages, including hypertension, obesity, and diabetes.^34,35^ Consequently, these risk factors are responsible for a large proportion of strokes at younger ages. In the INTERSTROKE study, the population attributable risk (PAR) of 10 traditional modifiable risk factors was 92.2% for stroke at ages ⩽ 55 years, with the highest PAR found for hypertension (49.7%) and lack of regular physical activity (35.3%).^21^ Hypertension had a higher PAR for ICH than ischemic stroke, but estimates for other risk factors were similar across aetiological subtypes of ischemic stroke.^22,32^ The role of traditional modifiable risk factors increased with age, but they still had a considerable contribution at the youngest end of the age spectrum. In INTERSTROKE, the PAR for hypertension was 23.3% at ages < 35 years.^22^

Time trends in the prevalence of traditional modifiable risk factors have been variable (Figure 3). While hypertension has been stable or decreasing in HICs, diabetes and obesity have become increasingly prevalent at younger ages over the past 30 years.^3,36–38^ An analysis of the GBD study suggested that high body mass index and high fasting plasma glucose were two of the three main contributors to the increase in disability adjusted life years attributable to ischemic stroke at younger ages between 1990 and 2019.^39^

In addition to their high prevalence, traditional modifiable risk factors tend to increasingly cluster in young patients with stroke, particularly in men. In a large Dutch cohort, 35% of patients aged < 55 years with ischemic stroke and 30% with ICH had two or more modifiable risk factors.^40^ In the Florida stroke registry, the proportion of patients with three or more traditional modifiable risk factors increased from 34.6% in 2014 to 41.9% in 2022 at ages 36–55 years, and from 10.9% to 16.4% at ages 18–35 years.^33^

Even though they are highly prevalent at younger ages, traditional modifiable risk factors are often unknown or undertreated.^41^ Young people are less likely to be aware and treated for their hypertension than older individuals, resulting in lower likelihood of blood pressure control in both high- and low-income settings.^42^ Similar age-related gaps in awareness and treatment have also been reported for diabetes.^43^ Part of this phenomenon might be related to current risk model-based management approaches in primary prevention.^44^ Given the heavy influence of age in existing models, it is perhaps not surprising that young people (particularly young women) are usually predicted to be below current treatment thresholds despite having elevated blood pressure or cholesterol levels. This may reduce the incentive to screen for risk factors and initiate primary prevention measures at younger ages. Conversely, management of risk factors may have preferentially improved at older ages (e.g. increased use of direct anticoagulants for atrial fibrillation), contributing to an age-specific divergence in stroke incidence.^45^

Data were sparse regarding traditional modifiable risk factors in young people with stroke from LMICs. The GOAL initiative showed that stroke patients in LMICs tended to be younger with fewer traditional modifiable vascular risk factors than in HICs, although hypertension and diabetes were more prevalent in Black (hypertension, 52.1%; diabetes, 20.7%) and Asian patients (hypertension 46.1%, diabetes, 20.9%).^46^ Ninety-percent of people with ischemic stroke aged < 50 years had hypertension in a multicentric study from Nigeria and Ghana, with two-thirds of events attributed to large artery-atherosclerosis or small-vessel disease.^47^ Different trends in the prevalence and control of traditional modifiable risk factors are likely responsible for some of the inequalities in young stroke incidence between HICs and LMICs. The Non-Communicable Diseases Risk Factor Collaboration showed that the age-standardized prevalence of hypertension increased between 1990 and 2019 in most LMICs compared to HICs.^48^ In the PURE cohort study, the prevalence of hypertension in participants aged < 50 years was similar in LMICs and HICs, but the rates of blood pressure treatment and control were lower in LMICs.^42^ Similar inequalities were reported for diabetes, with the lowest treatment rates in sub-Saharan Africa and South Asia.^49^ In addition to increasing rates of non-communicable diseases, LMICs still face an important burden of communicable diseases (e.g. rheumatic heart disease, human immunodeficiency virus), which may contribute to geographical disparities in stroke incidence.

Traditional modifiable risk factors are undoubtedly important contributors to stroke at younger ages. However, they might not be the main mechanistic driver for the observed time trends in stroke incidence. In OxVasc, the relative increase in stroke incidence at younger ages was also seen in people without hypertension, diabetes, or smoking, with the largest absolute increases seen in people with none of these risk factors and in cryptogenic stroke. There was also a simultaneous decrease in the incidence of myocardial infarction and peripheral vascular events at younger ages, suggesting that atherosclerosis was unlikely to be the main cause of the increase in young strokes.^3^

### Emerging risk factors?

Several non-traditional risk factors (Figure 3) have emerged as potential contributors to the unfavorable time trends in stroke incidence reported at younger ages. In this review, we examined some of these emergent risk factors and their potential contribution to stroke at younger ages. Further research is warranted to determine whether these associations were causal and specific to young patients.

Acute and chronic exposure to air pollution has been associated with an increased risk of stroke. The PURE study found a dose–response relationship between chronic exposure to outdoor particulate matter 2.5 and the rates of cardiovascular events during follow-up, with a stronger association (hazard ratio per 10 μg/m^3^ increase of particulate matter 2.5 = 1.07) and a higher PAR (19.6%) for stroke compared to myocardial infarction.^50^ Although no interaction with age was reported in PURE,^50^ a study from Israel suggested that the association between air pollution and stroke was only present in people aged < 55 years.^51^ Globally, the GBD study estimated that in 2021, ambient particulate matter pollution was the second largest contributor to stroke disability-adjusted life years (DALYs).^52^ Other environmental exposures have also been associated with stroke, including rising ambient temperatures across the globe and household pollution related to kerosene or solid fuel use in LMICs, but data were not stratified by age.^52,53^

Work-related factors have also been suggested to be potential risk factors for stroke at younger ages. A large individual patient data meta-analysis found that long working hours were more strongly associated with an increased risk of stroke than of myocardial infarction, with a dose-response relationship past 40 h per week.^54^ High levels of leisure sedentary time have been associated with stroke in the subset of young people with low levels of physical activity.^55^ Acute and chronic psychosocial stress may also be an important contributor. In the SECRETO study, self-perceived stress was associated with an increased risk of cryptogenic ischemic stroke at ages 18–49 years, even after adjustment for traditional vascular risk factors.^56^

Other emerging risk factors that have shown increasing trends in incidence and preferential associations with stroke at younger ages include prior autoimmune disease,^57^ depression,^58^ and illicit drug use.^59^ Early-onset cancer has also become more frequent in the 21^st^ century, reflecting the existence of shared risk factors with stroke at younger ages (e.g. obesity, poor diet), and potentially impacting the incidence of cancer-associated stroke.^12^

## Long-term impact: multifaceted questions

### Will I survive?

Among young patients, short term case fatality after stroke increases with age and is higher after ICH than ischemic stroke.^60^ Several population-based studies have found improvements over time.^60,61^ In England, case fatality after stroke at ages 20–54 years decreased by 7.2% between 2001 and 2010.^60^ However, other studies reported stagnating measures of case fatality.^62^ There are also significant regional disparities. In the GOAL initiative, 1-month case fatality after ischemic stroke at ages 18–50 years was 1.7% in HICs and 7.7% in LMICs.^46^ A hospital-based study in Mozambique (2005–2006) found that 28-day mortality was 19.1% and 53.6% at ages < 45 years after ischemic stroke and ICH, respectively.^63^

Data on long-term survival are more limited. In the FUTURE study, 30-day young stroke survivors had a 3.9-fold excess risk of death during the subsequent 20 years compared to the general population.^64^ The Swedish Inpatient Register (1987–2006) showed a decrease in 4-year mortality over time among 28-day young ischemic stroke survivors, but the latter remained at a sixfold higher risk of death than the general population.^65^

### Will I have another event?

Young stroke survivors are at high risk of recurrent stroke and other cardiovascular events. After an ischemic stroke, the 15-year cumulative risk of recurrent stroke was 16.4% in the FUTURE study^66^ and 19.1% in the Helsinki young stroke registry.^67^ Limited data are available regarding time trends in stroke recurrence. In the Swedish Inpatient Register, the 4-year rate of hospitalization for recurrent ischemic stroke decreased by 55% in males and 59% in females between 1987–1991 and 2002–2006.^68^ Conversely, a South Korean registry-based study found no improvement in the 1-year risk of recurrent stroke or TIA between 2011 and 2019.^62^ Overall, the risk of recurrent stroke is higher than of a subsequent cardiac or peripheral arterial event. In young people with atherosclerotic or cardioembolic stroke, both these risks are increased to a similar extent.^66,67^ Young stroke survivors are also at risk of bleeding. In the FUTURE study, the 10-year cumulative risk of minor or major bleeding was 21.8% after an ischemic stroke or TIA. Predictors of bleeding included female sex (mainly due to gynecological bleeds) and older age (40–49 years).^69^

### Will I make a full recovery?

Stroke at younger ages is associated with several adverse long-term outcomes. In a South Korean registry-based study, 32% of young patients with an ischemic stroke were dead or disabled (modified Rankin scale ⩾ 2) at 3 months, with no improvements in functional outcomes between 2011 and 2019.^62^ In a meta-analysis, 44% of young stroke survivors had residual cognitive impairment after at least 6 months of follow-up (excluding aphasia). Visuoconstruction abilities and processing speed were more frequently and severely affected.^70^ In the FUTURE study, after a mean follow-up of 9 years, 15% of young stroke survivors developed post-stroke epilepsy.^71^ Young stroke survivors are also at a threefold to fivefold higher risk of cancer diagnosis in the first year after the event compared to the general population.^72^ Moreover, psychosocial consequences are a frequent problem for these patients. In a recent meta-analysis, the pooled prevalence rates of post-stroke depression or anxiety symptoms were 31% and 39%, respectively.^73^ Only half of working-age adults are back at work 1-year post-stroke. Financial stress is common and is related to loss of income and out-of-pocket costs for health services.^74,75^ Unlike physical disability, impairment related to fatigue or cognitive impairment is often invisible to other people, limiting social reintegration and access to adequate support.^75^

## Projections and future directions

In 2021, the GBD study estimated that nearly 2.8 million people aged < 55 years developed an incident stroke globally. Eighty-nine percent of these events occurred in LMICs.^18^ The number of young stroke survivors is projected to significantly increase in the upcoming years.^76^ The American Heart Association estimated that from 2020 to 2050, stroke prevalence in the United States will rise from 0.8% to 2.6% at ages 20–44 years, and 3.8% to 5.6% at ages 45–64 years.^76^ An analysis of data from the GBD study projected that from 2020 to 2030, global stroke incidence rates will increase by a relative proportion of 11.8% at ages 20–39 years and 9.5% at ages 40–59 years.^77^

While ongoing monitoring of age-specific stroke incidence across the globe remains crucial, concerted efforts are urgently needed to reduce the projected burden of stroke at younger ages. Given the high and increasing prevalence of modifiable traditional risk factors at younger ages, improvement in their prevention and control could have a meaningful impact on young stroke incidence.^77^ Better risk prediction tools for young adults are also needed to facilitate the initiation and maintenance of primary prevention measures. Further research is still required to understand the mechanisms driving the age-specific divergence in stroke incidence rates, with a focus perhaps on the role of emerging risk factors and their potential synergistic effect with traditional risk factors and genetic susceptibility. Finally, while most studies reported data from HICs, research focused on LMICs is necessary to identify country-specific targets and strategies that could mitigate the impact of stroke at younger ages.

## Supplemental Material

sj-docx-1-wso-10.1177_17474930251362583 – Supplemental material for The rising incidence of stroke in the young: Epidemiology, causes and global impact Supplemental material, sj-docx-1-wso-10.1177_17474930251362583 for The rising incidence of stroke in the young: Epidemiology, causes and global impact by Ahmad Nehme and Linxin Li in International Journal of Stroke
