# Supplementary material for: The rising incidence of stroke in the young: Epidemiology, causes and global impact
Source: Int J Stroke. 2025 Jul 18;21(1):14–23. doi: 10.1177/17474930251362583 (PMC12743130; doi:10.1177/17474930251362583)
Supplement: sj-docx-1-wso-10.1177_17474930251362583 – Supplemental material for The rising incidence of stroke in the young: Epidemiology, causes and global impact [file sj-docx-1-wso-10.1177_17474930251362583.docx]

Web appendix additional references

Table 1

[1] Lisabeth LD, Brown DL, Zahuranec DB, et al. Temporal Trends in Ischemic Stroke Rates by Ethnicity, Sex, and Age (2000-2017): The Brain Attack Surveillance in Corpus Christi Project. *Neurology* 2021; 97:e2164-e2172.

[2] Madsen TE, Khoury JC, Leppert M, et al. Temporal Trends in Stroke Incidence Over Time by Sex and Age in the GCNKSS. *Stroke* 2020; 51:1070-1076.

[3] Scott CA, Li L and Rothwell PM. Diverging Temporal Trends in Stroke Incidence in Younger vs Older People: A Systematic Review and Meta-analysis. *JAMA Neurol* 2022; 79:1036-1048.

[4] Wafa HA, Wolfe CDA, Rudd A, et al. Long-term trends in incidence and risk factors for ischaemic stroke subtypes: Prospective population study of the South London Stroke Register. *PLoS Med* 2018; 15:e1002669.

[5] Appelros P. Secular Trends of Stroke Epidemiology in Örebro, Sweden, 2017 Compared to the Trends in 1999: A Population-Based Study. *Cerebrovasc Dis* 2019; 48:149-156.

[6] Aked J, Delavaran H, Norrving B, et al. Temporal Trends of Stroke Epidemiology in Southern Sweden: A Population-Based Study on Stroke Incidence and Early Case-Fatality. *Neuroepidemiology* 2018; 50:174-182.

[7] Béjot Y, Daubail B, Jacquin A, et al. Trends in the incidence of ischaemic stroke in young adults between 1985 and 2011: the Dijon Stroke Registry*. J Neurol Neurosurg Psychiatry* 2014; 85:509-513.

[8] Corso G, Bottacchi E, Giardini G, et al. Epidemiology of stroke in northern Italy: the Cerebrovascular Aosta Registry, 2004-2008. *Neurol Sci* 2013; 34:1071-1081.

[9] Correia M, Magalhães R, Felgueiras R, et al. Changes in stroke incidence, outcome, and associated factors in Porto between 1998 and 2011. *Int J Stroke* 2017; 12:169-179.

[10] Karantali E, Vemmos K, Tsampalas E, et al. Temporal trends in stroke incidence and case-fatality rates in Arcadia, Greece: A sequential, prospective, population-based study. *Int J Stroke* 2022; 17:37-47.

[11] Vibo R, Kõrv J and Roose M. The Third Stroke Registry in Tartu, Estonia: decline of stroke incidence and 28-day case-fatality rate since 1991. *Stroke* 2005; 36:2544-2548.

[12] Krishnamurthi RV, Barker-Collo S, Parag V, et al. Stroke Incidence by Major Pathological Type and Ischemic Subtypes in the Auckland Regional Community Stroke Studies: Changes Between 2002 and 2011. *Stroke* 2018; 49:3-10.

[13] Olindo S, Chausson N, Mejdoubi M, et al. Trends in incidence and early outcomes in a Black Afro-Caribbean population from 1999 to 2012: Etude Réalisée en Martinique et Centrée sur l'Incidence des Accidents Vasculaires Cérébraux II Study. *Stroke* 2014; 45:3367-3373.

[14] Kita Y, Turin TC, Ichikawa M, et al. Trend of stroke incidence in a Japanese population: Takashima stroke registry, 1990-2001. *Int J Stroke* 2009; 4:241-249.

[15] Minelli C, Cabral NL, Ujikawa LT, et al. Trends in the Incidence and Mortality of Stroke in Matão, Brazil: The Matão Preventing Stroke (MAPS) Study. *Neuroepidemiology* 2020; 54:75-82.

Figure 3

[1] Worldwide trends in hypertension prevalence and progress in treatment and control from 1990 to 2019: a pooled analysis of 1201 population-representative studies with 104 million participants. Lancet 2021; 398:957-980.

[2] Worldwide trends in diabetes prevalence and treatment from 1990 to 2022: a pooled analysis of 1108 population-representative studies with 141 million participants. *Lancet* 2024; 404:2077-2093.

[3] Repositioning of the global epicentre of non-optimal cholesterol. *Nature* 2020; 582:73-77.

[4] Ng M, Gakidou E, Lo J, et al. Global, regional, and national prevalence of adult overweight and obesity, 1990-2021, with forecasts to 2050: a forecasting study for the Global Burden of Disease Study 2021*. Lancet* 2025; 405:813-838.

[5] Dai X, Gakidou E and Lopez AD. Evolution of the global smoking epidemic over the past half century: strengthening the evidence base for policy action. *Tob Control* 2022; 31:129-137.

[6] Manthey J, Shield KD, Rylett M, et al. Global alcohol exposure between 1990 and 2017 and forecasts until 2030: a modelling study. *Lancet* 2019; 393:2493-2502.

[7] Imamura F, Micha R, Khatibzadeh S, et al. Dietary quality among men and women in 187 countries in 1990 and 2010: a systematic assessment. *Lancet Glob Health* 2015; 3:e132-e142.

[8] Strain T, Flaxman S, Guthold R, et al. National, regional, and global trends in insufficient physical activity among adults from 2000 to 2022: a pooled analysis of 507 population-based surveys with 5·7 million participants. *Lancet Glob Health* 2024; 12:e1232-e1243.

[9] Yu W, Ye T, Zhang Y, et al. Global estimates of daily ambient fine particulate matter concentrations and unequal spatiotemporal distribution of population exposure: a machine learning modelling study. *Lancet Planet Health* 2023; 7:e209-e218.

[10] Bennitt FB, Wozniak S, Causey K, et al. Global, regional, and national burden of household air pollution, 1990-2021: a systematic analysis for the Global Burden of Disease Study 2021. *Lancet*; 405:1167-1181.

[11] Romanello M, Napoli CD, Green C, et al. The 2023 report of the Lancet Countdown on health and climate change: the imperative for a health-centred response in a world facing irreversible harms. *Lancet* 2023; 402:2346-2394.

[12] Pega F, Náfrádi B, Momen NC, et al. Global, regional, and national burdens of ischemic heart disease and stroke attributable to exposure to long working hours for 194 countries, 2000-2016: A systematic analysis from the WHO/ILO Joint Estimates of the Work-related Burden of Disease and Injury. *Environ Int* 2021; 154:106595.

[13] Chen S, Huang W, Zhang M, et al. Dynamic changes and future trend predictions of the global burden of anxiety disorders: analysis of 204 countries and regions from 1990 to 2021 and the impact of the COVID-19 pandemic. *EClinicalMedicine* 2025; 79:103014.

[14] Yang J, Zhang L, Yang C, et al. Global, Regional, and National Epidemiology of Depression in Working‐Age Individuals, 1990–2019. *Depression and Anxiety* 2024. DOI: 10.1155/2024/4747449.

[15] Zhang F, Cui Y, Gao X. Time trends in the burden of autoimmune diseases across the BRICS: an age-period-cohort analysis for the GBD 2019. *RMD Open* 2023; 9:e003650.

[16] Conrad N, Misra S, Verbakel JY, et al. Incidence, prevalence, and co-occurrence of autoimmune disorders over time and by age, sex, and socioeconomic status: a population-based cohort study of 22 million individuals in the UK*. Lancet* 2023; 401:1878-1890.

[17] Bender RG, Sirota SB, Swetschinski LR, et al. Global, regional, and national incidence and mortality burden of non-COVID-19 lower respiratory infections and aetiologies, 1990-2021: a systematic analysis from the Global Burden of Disease Study 2021. *Lancet Infectious Diseases* 2024; 24:974-1002.

[18] Chen A, Zou M, Fan M, et al. Time trends and associated factors of global burden due to drug use disorders in 204 countries and territories, 1990-2019. *Drug Alcohol Depend* 2022. DOI: 10.1016/j.drugalcdep.2022.109542.

[19] Zhao J, Xu L, Sun J, et al. Global trends in incidence, death, burden and risk factors of early-onset cancer from 1990 to 2019. *BMJ Oncol* 2023; 2:e000049.
